# The Strengths and Difficulties Questionnaire as a Predictor of Parent-Reported Diagnosis of Autism Spectrum Disorder and Attention Deficit Hyperactivity Disorder

**DOI:** 10.1371/journal.pone.0080247

**Published:** 2013-12-03

**Authors:** Ginny Russell, Lauren R. Rodgers, Tamsin Ford

**Affiliations:** 1 Institute of Health Services Research, University of Exeter Medical School, Exeter, United Kingdom; 2 NIHR CLAHRC for the South West Peninsula (PenCLAHRC), University of Exeter Medical School, Exeter, United Kingdom; Birkbeck, University of London, United Kingdom

## Abstract

The Strengths and Difficulties Questionnaire (SDQ) is widely used as an international standardised instrument measuring child behaviour. The primary aim of our study was to examine whether behavioral symptoms measured by SDQ were elevated among children with autism spectrum disorder (ASD) and attention deficit hyperactivity disorder (ADHD) relative to the rest of the population, and to examine the predictive value of the SDQ for outcome of parent-reported clinical diagnosis of ASD/ADHD. A secondary aim was to examine the extent of overlap in symptoms between children diagnosed with these two disorders, as measured by the SDQ subscales. A cross-sectional secondary analysis of data from the Millennium Birth Cohort (n = 19,519), was conducted. Data were weighted to be representative of the UK population as a whole. ADHD or ASD identified by a medical doctor or health professional were reported by parents in 2008 and this was the case definition of diagnosis; (ADHD n = 173, ASD n = 209, excluding twins and triplets). Study children's ages ranged from 6.3–8.2 years; (mean 7.2 years). Logistic regression was used to examine the association between the parent-reported clinical diagnosis of ASD/ADHD and teacher and parent-reported SDQ subscales. All SDQ subscales were strongly associated with both ASD and ADHD. There was substantial co-occurrence of behavioral difficulties between children diagnosed with ASD and those diagnosed with ADHD. After adjustment for other subscales, the final model for ADHD, contained hyperactivity/inattention and impact symptoms only and had a sensitivity of 91% and specificity of 90%; (AUC) = 0.94 (95% CI, 0.90–0.97). The final model for ASD was composed of all subscales except the ‘peer problems’ scales, indicating of the complexity of behavioural difficulties that may accompany ASD. A threshold of 0.03 produced model sensitivity and specificity of 79% and 93% respectively; AUC = 0.90 (95% CI, 0.86–0.95). The results support changes to DSM-5 removing exclusivity clauses.

## Introduction

The Strengths and Difficulties Questionnaire (SDQ) is a brief dimensional measure of psychopathology among children aged 4–16 that has been widely adopted in both research and in clinical practice [1]. The instrument is composed of 25 items that ask about behavioral attributes of the child and are combined to form five subscales (composed of 5 items each). The subscales measure emotional symptoms, conduct problems, hyperactivity/inattention, peer relationships, and prosocial behavior. There are parallel versions of the SDQ that collect the same data from parents, teachers and young people aged 11 or over. A supplemental ‘impact’ subscale measures chronicity, distress, social impairment, and burden to others, which provides useful additional information for clinicians and researchers [2].

The SDQ has been used in in clinical practice as a screening and/or assessment tool by both school psychologists [3] and clinicians [2], [4], [5]. It is also used extensively in research studies throughout Europe [6]–[8] the USA [9], [10], Asia [5], [11]–[13] and Africa [13]. To date, the SDQ has received over 3,000 research citations and this number is growing, particularly as many on-going longitudinal birth cohorts have used the SDQ for over a decade as a repeated measure of child behaviour [7], [14], [15].

Woerner and colleagues [8] reviewed non-European studies that psychometrically evaluated the SDQ, applied it to screen for behaviour disorders, or employed its parent-, teacher- or self-rated versions as research tools. They found experience gained with the SDQ in other continents has supported European evidence of good psychometric properties and clinical utility. They note that worldwide usage of the SDQ is expected to increase in the future, although reporting by different participants is context-dependent and this limits the reliability of cross-cultural comparisons [7], [16]. Despite these reservations, the SDQ has been successfully used to make comparisons of child behaviour across age and culture [17].

Various studies have examined the utility of the SDQ as a screening device in predicting childhood psychiatric cases [18]–[20] although few have looked at SDQ as a screen for specific disorders. In a UK community-based sample, multi-informant ratings [parents, teachers and older children] identified individuals with specific psychiatric diagnoses [21]. Sensitivity was over 70% for identifying conduct and hyperactivity disorders, but the instrument had poor discrimination (<30%) for emotional disorders in this general population sample. Varying results are most likely due to the heterogeneity of symptoms of childhood emotional disorders which have a wide range of symptoms, only some of which are captured by the five questions about emotional difficulties in the SDQ. Notably, there are no questions that directly relate to the triad of difficulties that comprise the autism spectrum, although indirectly, social skills can be inferred from the prosocial and peer relationship subscales. In contrast, there are five questions each on the more homogenous area of difficulties with attention/ hyperactivity.

Goodman and colleagues [21] developed an SDQ algorithm that combines teacher, parent and child reports, to predict various disorders, including ‘Probable Hyperactive Disorder’ (PHD) in children. The PHD algorithm uses a combination of informants for SDQ scores on the hyperactivity/inattention and impact subscales [18], [22]. Multiple informants are required because symptoms must be present across multiple settings if ADHD is to be diagnosed [23], [24]. Ullebo and colleagues [20] tested the PHD algorithm and found that it had an acceptable sensitivity for the ADHD combined phenotype. They recommended that bespoke cut-offs should be developed according to the purpose of its application to research. Brøndbo and colleagues [25] cautioned against use of the PHD algorithm as a screening instrument for ADHD in the clinic because of the large number of false positives identified.

According to the International Classification of Diseases (ICD-10), for a diagnosis of ASD to be made, children must display impairments in social interactions and communication, as well as restricted interests and repetitive behaviour [23]. Iizuka and colleagues [26] examined the co-occurrence of behavioural symptoms of high-functioning ASD and ADHD using the SDQ subscales in Japan. Core symptoms of ASD include social and communication impairments and, as expected, the two SDQ subscales that measure aspects of social interaction- peer problems and prosocial behaviour- were associated with ASD in particular. The study found elevated levels of peer problems and emotional difficulties, and fewer prosocial behaviours among the ASD group compared to those children with ADHD, whilst higher levels of hyperactivity and more conduct problems were reported for children with ADHD. A large and growing literature has demonstrated that ADHD symptoms are relatively common among children and adults with ASD and vice-versa [27]–[33]. Recently, some researchers have queried whether ASD and ADHD should be considered as different manifestations of one overarching disorder [33], [34]. Currently, the diagnostic criteria for childhood disorders laid out in ICD-10 contain an exclusivity clause that does not allow ADHD to be diagnosed if pervasive developmental disorder (including ASD) is present, although the exclusivity clause has been dropped in the new version of the Diagnostic and Statistical Manual of Mental Disorders (DSM-5), in which ASD is listed as a condition which is commonly comorbid with ADHD [24].

Given the wide and on-going use of the SDQ in research on developmental disorders, we sought to clarify the predictive power of the SDQ subscales in the identification of parent-reported clinical diagnosis of two specific disorders: ADHD and ASD. The primary aim of our study was to examine whether all behavioral symptoms measured by SDQ were elevated in children with ASD and ADHD relative to the rest of the population, and the utility of the SDQ as an indicator of these disorders. A secondary aim was to examine the extent to which symptoms co-occurred in children diagnosed with ASD or ADHD. We hypothesised hyperactivity/inattention symptoms would predict clinical diagnosis of ADHD, and prosocial and peer relationship problems would predict ASD. This study therefore assesses the utility of the SDQ in identifying these disorders.

## Methods

### Ethics Statement

Information was gathered from the sample, the first Millennium Cohort Study (MCS) survey when children were 9 months old, and three, five and seven years of age: four sweeps of data collection [35]. Informed written consent was obtained at each stage of the study from parents for their participation and the participation of their child (ren); the MCS ethical review gives details [36]. Written consent was also obtained for gathering information from health, education and economic records and to contact teachers. The data were analyzed anonymously, with researchers having no access to participant identities. Identities are protected by the curators of the MCS. Additional ethical approval for the analysis reported here was granted by the Peninsula Medical School Ethics committee.

### Design

Our study sought to clarify the predictive power of the SDQ subscales in the identification of parent-reported diagnosis of ASD and ADHD using logistic regression models. This was compared to the predictive power of the PHD algorithm already in existence [21]. A secondary aim was to examine the extent of overlap in symptoms between children diagnosed with these two disorders, as measured by the SDQ subscales, in order to inform the debate about revisions to diagnostic criteria.

### Sample

The Millennium Cohort Study (MCS) is a UK-representative birth cohort study that used a disproportionate stratified cluster sampling design [35], [37]. Children born between 1^st^ September 2000 and 11^th^ January 2002 and listed on the Child Benefit Records were eligible for the study. Child Benefit was a financial benefit payable to all parents of UK children at this time, with near universal take up. Data were first collected when children were 9 months old (1^st^ wave), further data were recorded concerning the children's health and development when the children were 3 years old (2^nd^ wave), 5 years old (3^rd^ wave) and 7 years old (4^th^ wave). Within the total MCS cohort of 19, 519 children, the current study outcomes, ASD and ADHD status, were recorded for 14, 043 children at wave 4 (over 70%). The MCS provides appropriate standardised weightings to adjust for the effect of attrition and oversampling, making these results representative of the UK population as a whole. Details of sampling design and weighting calculations are documented in detail elsewhere [37].

### Outcome measures

The case definition of the two conditions was based on responses to an MCS question duplicated from the US National Health Interview Survey questionnaire reported in previous studies [38]. Parents or carers were asked in face-to-face interviews if a doctor or health professional had identified childhood ADHD or ASD. Consistent with other studies using these data [39], families with twins or triplets where other siblings participated were excluded (252 twins, 11 triplets) as both diagnoses have a high heritability. Parent-reported ASD and/or ADHD diagnosis was recorded for 14,043 children in 2008/9 with the wording of the following questions read out verbatim:

- *Has a doctor or health professional ever told you that (sample child) had attention deficit hyperactivity disorder (ADHD)*
- *Has a doctor or health professional ever told you that (sample child) had autism, Asperger's syndrome or autistic spectrum disorder?*

Families at wave 4 whose study children were seven years old, who responded with positive or negative answers to the above questions, were included. Families who answered ‘don’t know' or refused to answer were excluded from the analysis (*n* = 30 ASD, *n* = 44 ADHD, of these, *n* = 17 refused/don't know in both categories). We took this measure to represent a clinical diagnosis of disorder in line with other studies [38], [40], [41]. In total, from this sample, 173 children had reportedly been identified with ADHD and 209 had a parent-reported ASD diagnosis by age 7. Forty-four children had a co-morbid diagnosis of ASD and ADHD, and were retained in both outcome groups.

### Independent variables

The SDQ is composed of 25 items that ask about behavioural attributes of the child and are combined to form five subscales (composed of 5 items each). The emotional symptoms subscale contains items that ask about fears, worries, misery, nerves and somatic symptoms, the conduct problems subscale inquires about tantrums, obedience, fighting, lying and stealing, and the hyperactivity/inattention subscale covers restlessness, fidgeting, concentration, distractibility and impulsivity. The peer relationships subscale items include questions about popularity, victimization, isolation, friendship and ability to relate to children as compared to adults, and the prosocial subscale covers consideration of others, ability to share, kindness to younger children, and helpfulness when other children are distressed and willingness to volunteer to comfort. For all the subscales except the prosocial subscale, high scores indicate difficulties. As the prosocial items ask about the presence of prosocial behaviour, the subscale measures the strengths of the child in this area, and increasing scores represent increasingly prosocial behaviour, unlike the other sub-scales where increasing score represents increasing impairment. In all cases, answer options for each item are: ‘Not true’ ‘Somewhat true’ or ‘Certainly true’, and these are scored 0, 1 or 2, giving a total score out of a possible 10 for each subscale. A further ‘impact’ subscale measures the impact of any difficulties on carers and the children themselves in terms of chronicity, distress, social impairment, and burden to others. This is again scored 0–10 with increasing impact producing a higher score. More details about the SDQ, the probable hyperactivity disorder (PHD) algorithm, normative data, background research and how the subscales are scored are available at the SDQ website (www.sdqinfo.org).

SDQ scores for each subscale had been taken for the entire cohort at wave 4 from both parent and teacher informants. Both were added to models, since clinical identification of the disorders should be documented as causing impairment across settings (for example, home and school). Several studies have stressed the need for information from multiple informants when rating symptoms of a child psychiatric disorder [42].

### Analysis

The ASD, ADHD and general population were compared on SDQ subscale scores. Box plots were provided for teacher and parent report of behaviour separately to illustrate how the three groups (ASD, ADHD and general population) differed in SDQ scores. Children reported as having both diagnoses (n = 44) were included in both ASD and ADHD groups.

Logistic regression (LR) established the odds of diagnosis of ASD/ADHD using SDQ subscales as independent variables. Parent and teacher ratings of behaviour were treated as separate covariates. The odds ratios (OR) from the analyses indicate that the relative increase in odds of being identified with ASD/ADHD corresponded to a one-point increase in the SDQ subscales. All the sub-scales bar the prosocial scale measure impairment, therefore the reciprocal of the odds ratios for the prosocial scores was used to fit conceptually with the rest of the model. This means that for all SDQ subscales, an odds ratio greater than 1 represents greater prediction of diagnosis as children's difficulties increase. Unadjusted logistic regression models were fitted in which just one predictor at a time was included. Multivariable (adjusted) logistic regression models were then fitted in which predictors significant at the 10% level in the unadjusted analyses were included as covariates. Estimates from LR were weighted to take account of the disproportionate stratified sample of electoral wards and attrition/non-response by the 4^th^ wave when the study outcomes were measured, making the sample representative of the UK population [37]. LR was then used to derive separate models for ASD and for ADHD respectively, composed of the SDQ subscales that remained significant at 10% levels after adjustment for other subscales. Final models were composed of subscales that remained significantly associated with outcome at 10% levels after adjustment for other behaviours. The sensitivity (percentage of children with diagnosis correctly identified as such) and specificity (probability that a test result will be negative when the disease is not present or true negative rate, expressed as a percentage) of the final models were examined using Receiver Operating Characteristic (ROC) curves. The area under the curve (AUC) is a measure of how well the model can identify children with disorder. The Youden Index [43] is used to calculate the optimal values for sensitivity and specificity; it determines a threshold that will maximise the difference between true positive and false positive rates. For this threshold, the positive predictive value was derived for each model. In the case of ADHD, the sensitivity and specificity were compared to the cut-offs for ‘Probable Hyperactivity Disorder’ algorithm [21].

## Results

**Figure 1:**
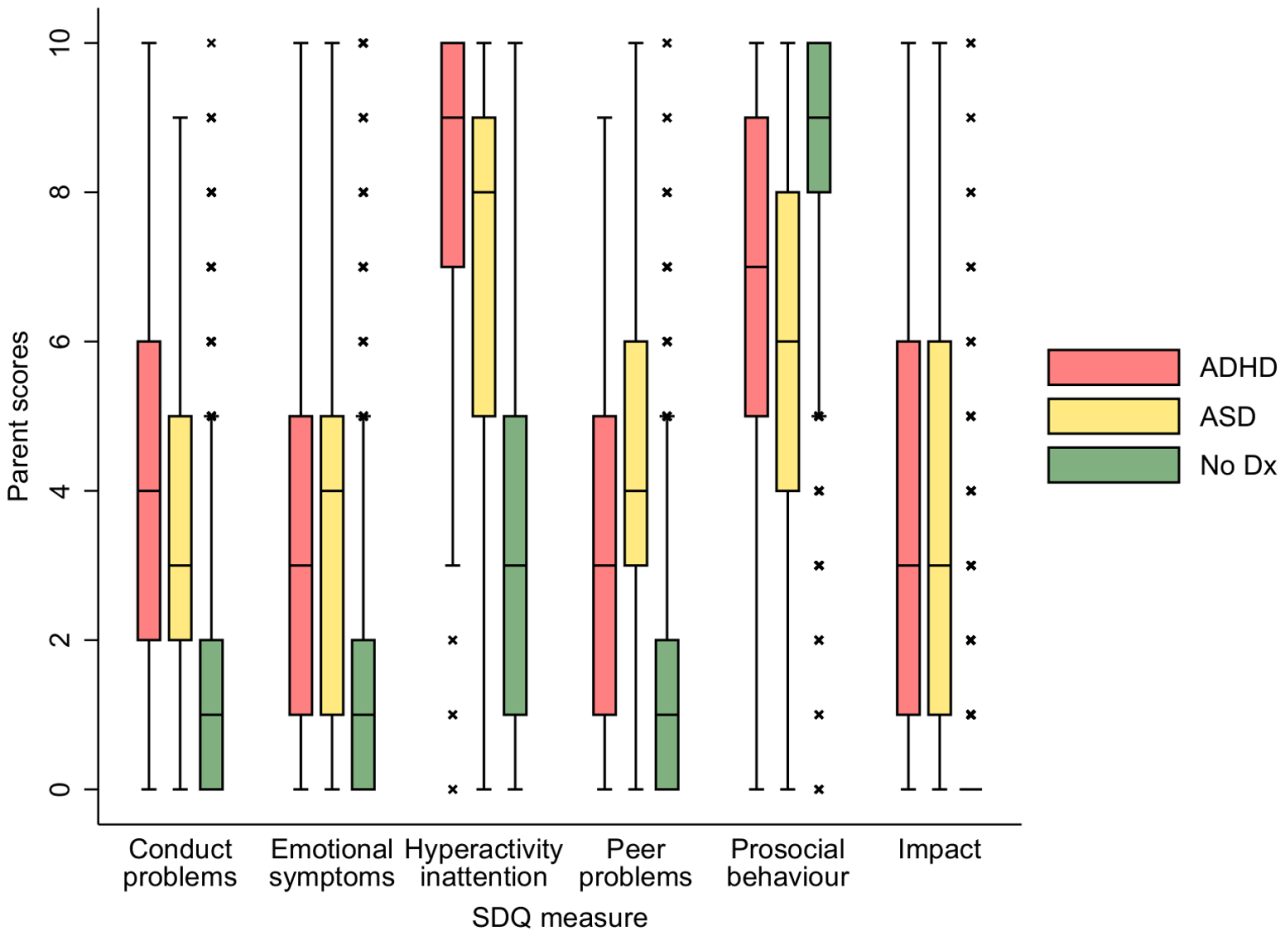
Box plots for parent report of SDQ subscales across three groups: ASD diagnosis, ADHD diagnosis and neither diagnosis. Diagnosis: dx Increasing score reflects increased impairment in all sub-scales except prosocial scores which measure strengths.

**Figure 2:**
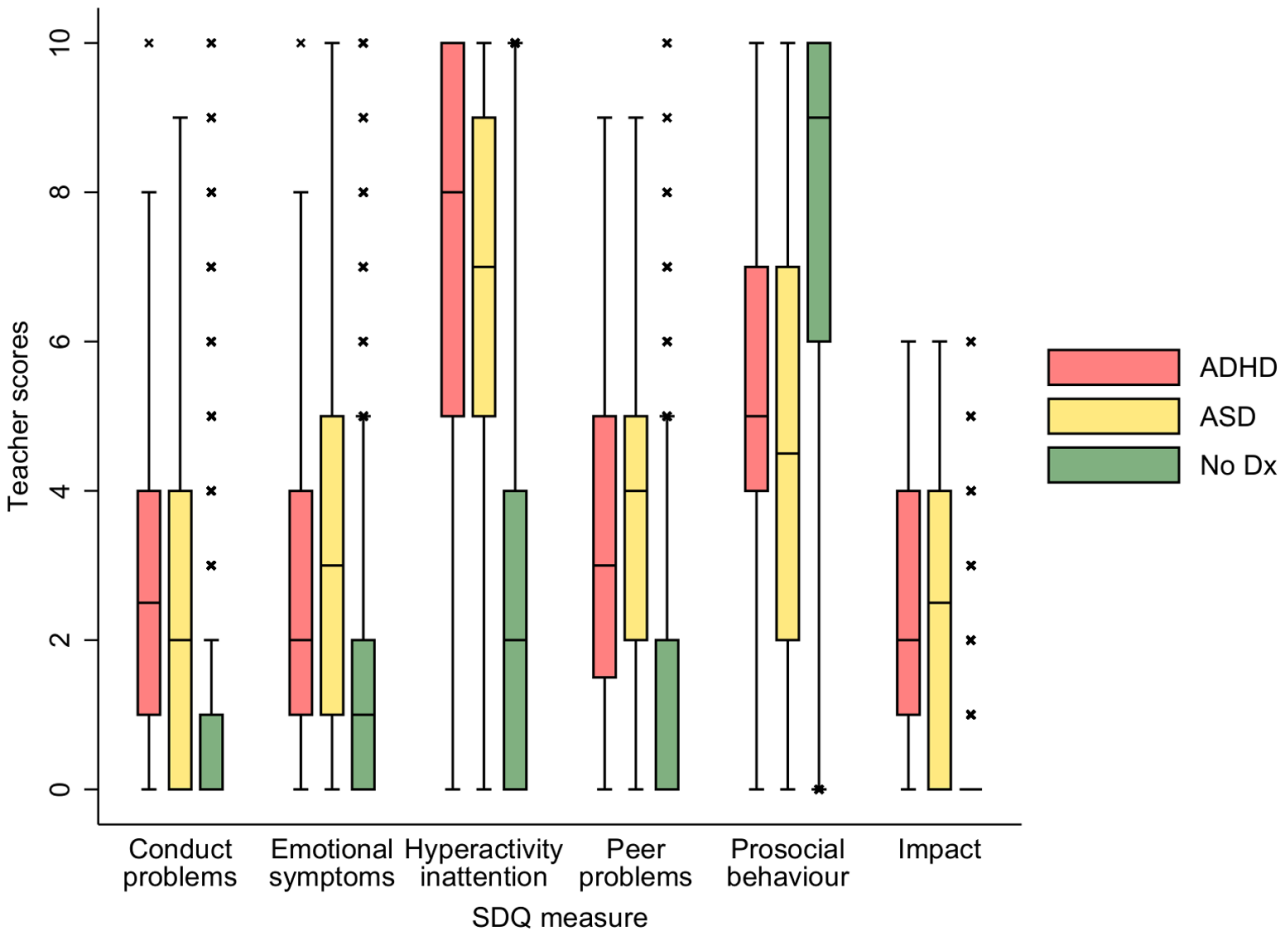
Box plots for teacher report of SDQ subscales across three groups: ASD diagnosis, ADHD diagnosis and neither diagnosis. Diagnosis: dx Increasing score reflects increased impairment in all sub-scales except prosocial scores which measure strengths.

For 96.7% of families participating, the main respondent on the outcome measure of ASD or ADHD was the child's mother. At the birth of the child, mothers had a mean age of 28 years (range 13 to 48 years), and over 99% were resident at home with the study child all of the time. The mean child age when outcome measures were taken was 7.2 years (SD = 0.2; range, 6.3 to 8.2). Figures 1 and 2 illustrate the demographic profile of the sample, giving descriptive statistics for parent and teacher-rated SDQ subscales for children with ASD, those with ADHD and those with neither diagnosis. Clear differences are observed between the children with neither diagnosis (no dx) and children with ADHD/ASD. The figures illustrate differences in the distribution of scores between ASD children and those with ADHD but also substantial overlap. The inter-rater reliability between parent and teacher scores was low to medium, values of the weighted kappa coefficient ranged from 0.24 for the emotional symptoms sub-score (95% CI 0.22–0.27) to 0.47 for hyperactivity/inattention scores (95% CI 0.45−0.47).

As Figure 1 and Figure 2 illustrate, children with ADHD and ASD diagnoses had substantially more impaired behaviour at age 7 than other participating children without either of these diagnoses on every SDQ sub-scale. Hyperactivity scores were particularly high in both diagnosed samples compared to those of the rest of the population. Impact scores were also higher and prosocial skills were lower in both diagnosed groups according to both informants.

Table 1 reports the results of LR for the outcome of ADHD. These results confirm that all subscales were significantly associated with diagnosis of ADHD, and reflect greater impairment across the range of behaviours measured by the SDQ instrument. After adjustment for the other SDQ subscales, only teacher and parent-reported hyperactivity/inattention subscales and parent-reported impact remained significantly associated with ADHD diagnosis. A threshold of 0.02 from the model yielded the optimal sensitivity and specificity values of 91% and 90% respectively. The positive predictive value (PPV) was low at 12%, which is to be expected in a population based sample screening for rare disorders comprising young children. The Area Under the Curve (AUC)  = 0.94 (95% CI, 0.90–0.97) shows the model is a good fit. The PHD algorithm [21] produces a sensitivity of just 30%, but a specificity of 98% for the ‘probable hyperactivity disorder’ category. The positive predictive power was also fairly low at 27%. Examples of scores that exceed the threshold for this model using optimal values are given in the supporting information in Table S1.

**Table 1:** Models showing SDQ sub-scales as predictors with ADHD diagnosis as outcome for children from Milenium Cohort at age 7.

Variable	Unadjusted n	Unadjusted OR & 95% CI	Unadjusted p	Adjusted n	Adjusted OR & 95% CI	Adjusted p^1^	Final n	Final OR & 95% CI^3^	Final p
Emotion Parent	13082	1.50 (1.40,1.60)	<0.001	8133	1.11 (0.96,1.28)	0.17			
Emotion Teacher	8511	1.25 (1.16,1.34)	<0.001	8133	0.89 (0.77,1.04)	0.13			
Conduct Parent	13111	1.85 (1.72,1.99)	<0.001	8133	1.07 (0.93,1.23)	0.37			
Conduct Teacher	8514	1.49 (1.41,1.58)	<0.001	8133	0.93 (0.79,1.09)	0.36			
**Hyper Parent**	13061	2.17 (1.95,2.42)	<0.001	8133	1.56 (1.34,1.83)	<0.001	8277	1.56 (1.35,1.80)	<0.001
**Hyper Teacher**	8512	1.60 (1.48,1.72)	<0.001	8133	1.19 (1.06,1.35)	0.003	8277	1.22 (1.11,1.34)	<0.001
Prosocial Parent^2^	13116	0.65(0.59,0.70)	<0.001	8133	0.99 (0.87,1.13)	0.87			
Prosocial Teacher^2^	8510	0.68(0.63,0.72)	<0.001	8133	1.05 (0.92,1.19)	0.46			
Peer Parent	13094	1.68 (1.56,1.80)	<0.001	8133	0.93 (0.81,1.07)	0.32			
Peer Teacher	8511	1.53 (1.42,1.64)	<0.001	8133	1.09 (0.93,1.28)	0.28			
**Impact Parent**	12958	2.17 (1.96,2.41)	<0.001	8133	1.56 (1.37,1.78)	<0.001	8277	1.63 (1.45,1.84)	<0.001
Impact Teacher	8404	2.19 (1.98,2.41)	<0.001	8133	1.12 (0.92,1.36)	0.25			

1 Adjusted models include subscales significant at 10% levels. 2.For prosocial scores the reciprocal of the odds ratios.

Is presented to fit conceptually with the rest of the model, i.e. greater OR = greater association with ADHD. 3. Model constant 0.0002 on odds ratio scale.

LR was also used to explore the predictive value of the subscales for ASD (Table 2).

**Table 2:** Models of SDQ sub-scales as predictors with ASD diagnosis as outcome for children from Milenium Cohort at age 7.

Variable	Unadjusted n	Unadjusted OR & 95% CI	Unadjusted p	Adjusted n	Adjusted OR & 95% CI	Adjusted p^1^	Final n	Final ASD OR & 95% CI^3^	Final p
**Emotion Parent**	13127	1.52 (1.41,1.64)	<0.001	8162	1.15 (1.00,1.33)	0.04	8180	1.16 (1.01,1.33)	0.04
Emotion Teacher	8536	1.38 (1.29,1.48)	<0.001	8162	1.05 (0.93,1.20)	0.42			
Conduct Parent	13155	1.63 (1.53,1.74)	<0.001	8162	0.92 (0.80,1.06)	0.26			
**Conduct Teacher**	8539	1.41 (1.33,1.50)	<0.001	8162	0.83 (0.74,0.95)	0.005	8180	0.86 (0.76,0.96)	0.01
**Hyper Parent**	13105	1.75 (1.63,1.88)	<0.001	8162	1.18 (1.04,1.33)	0.009	8180	1.15 (1.03,1.30)	0.02
**Hyper Teacher**	8537	1.48 (1.38,1.58)	<0.001	8162	1.10 (1.00,1.22)	0.06	8180	1.11 (1.01,1.23)	0.03
**Prosocial Parent^2^**	13159	0.54 (0.49,0.59)	<0.001	8162	1.24 (1.10,1.41)	0.001	8180	1.25 (1.11,1.42)	<0.001
Prosocial Teacher^2^	8535	0.64 (0.59,0.70)	<0.001	8162	1.10 (0.97,1.24)	0.14			
Peer Parent	13136	1.94 (1.80,2.09)	<0.001	8162	1.04 (0.88,1.23)	0.62			
Peer Teacher	8536	1.65 (1.53,1.77)	<0.001	8162	1.06 (0.91,1.23)	0.46			
**Impact Parent**	13004	2.11 (1.93,2.30)	<0.001	8162	1.51 (1.30,1.75)	<0.001	8180	1.53 (1.35,1.72)	<0.001
**Impact Teacher**	8430	2.31 (2.10,2.54)	<0.001	8162	1.25 (1.02,1.55)	0.04	8180	1.44 (1.22,1.71)	<0.001

1 Adjusted models include subscales significant at 10% levels. 2.For prosocial scores the reciprocal of the odds ratios.

Is presented to fit conceptually with the rest of the model, i.e. greater OR = greater association with ASD. 3. Model constant 0.113 on odds ratio scale.

Again all the SDQ subscales were significant in unadjusted analysis. After adjustment for interdependencies between subscales, several still remained significantly associated with the outcome of ASD at 10% levels. These were the impact and hyperactivity subscales from both raters, and the prosocial and emotional symptoms scores rated by parents. The measures with the largest effect were the parent-rated subscales of the prosocial behaviour and impact subscales. Peer problems from either rater did not appear in the final model. A threshold of 0.03 produced the optimal values for model sensitivity and specificity of 79% and 93% respectively; AUC = 0.90, (95% CI, 0.86–0.95). The PPV was again low at 18%. Examples of scores that exceed the threshold for this ASD model using optimal values are given in the supporting information in Table S2. Table 3 shows the threshold, sensitivity and specificity for higher PPVs for both the ASD and ADHD model, illustrating the varying sensitivity, specificity and predictive power of the model at various threshold settings.

**Table 3:** Varying sensitivity, specificity and positive predictive values for ASD and ADHD models derived from Millennium Cohort Data.

PPV	ADHD			ASD		
	Threshold	Sensitivity	Specificity	Threshold	Sensitivity	Specificity
20%	0.05	81%	95%	0.04	76%	94%
30%	0.13	63%	98%	0.08	66%	97%
40%	0.39	34%	99%	0.17	53%	98%
50%	0.74	15%	100%	0.28	47%	99%
60%	0.92	5%	100%	0.57	29%	100%

## Discussion

The prevalence of ASD and ADHD was not the focus of this paper: we have written about this elsewhere [44]. The low prevalence of parent-reported ADHD diagnosis is consistent with other UK studies [45] and studies in Scandinavia [46]. The reported prevalence of ASD diagnosis is high compared to previous estimates; which may reflect the increasing use of the ASD label in the UK, a trend that has also been identified in other studies. Results showed elevated behavioral difficulties in multiple domains for both groups with parent-reported diagnoses, and suggests that many behavioral problems are shared by children diagnosed with ASD and those diagnosed with ADHD.

Despite the exclusivity clause in the current ICD-10 diagnostic classification systems, there was a high proportion of dual diagnosis in the two conditions: 23% of children with ADHD had a diagnosis of ASD, and 21% with ASD had identified ADHD. Several other recent studies [29]–[33] also suggest that children with ASD and ADHD often share symptoms of hyperactivity and other behavioural difficulties. ADHD symptoms are relatively common in children and adults with autistic-type symptoms; autism-type symptoms/ behaviours may be less common in children with ADHD [27], [28], [30], [31]. Our findings of elevated behavioral difficulties indicative of both conditions in both diagnosed groups support change to the diagnostic criteria to allow ASD and ADHD to be diagnosed in the same individual. Our findings suggest that this already relatively common in practice, so removal of the exclusivity clauses would eliminate unnecessary tension between clinical practice and diagnostic rules.

After adjustment for other subscales in multivariable models, the final model for ADHD was composed of the hyperactivity/inattention and impact symptoms only. This finding is highly predictable and as initially hypothesised, although the selection biases inherent in obtaining a clinical diagnosis may have clouded the relationship. Although the findings suggest that ADHD symptoms are also relatively common in children with ASD and vice-versa, in line with findings from other studies [27]–[33], the results do not support the argument that ASD and ADHD should be considered as different manifestations of one overarching disorder [33], [34].

In our study LR models, after statistical adjustment for interdependencies between different types of behavioral problems, a distinctive symptom profile emerged for ADHD based on hyperactivity and impact sub-scales, but not for ASD. The finding provides evidence to support the assertion of Nicalsen et al. [22] that the SDQ hyperactivity-inattentive subscale shows good agreement with the diagnostic criteria for attention-deficit hyperactivity disorder, as it was intended to do. Our adjusted results suggest that children with ADHD have focused problems of hyperactivity/inattention. A combined model derived from both parent and teacher hyperactivity and impact scores is a good predictor of diagnosis of ADHD, identifying up to 91% of children with parent-reported clinical diagnosis of ADHD. The models developed using the MCS data and the Goodman PHD algorithm differ in two ways. First, the MCS model did not find impact on teachers to be a significant predictor of ADHD. This contradicts arguments of many socially orientated researchers who suggest that ADHD is partially constructed in response to the need for compliance at school [47]. Others have observed that ADHD is more likely to be identified in tandem with disruption to the classroom [48]. MCS data suggests for teachers, presence of inattention and hyperactivity alone is enough to indicate ADHD. One partial explanation could be that naming the condition: ADHD being diagnosed; minimises teacher ratings of impact.

The second main difference is that cut-offs (e.g. for identifying 91% of children with disorder) were not fixed as are those in the PHD model. This is consistent with the findings of Ullebo and colleagues [20], who conclude, and the ROC curves demonstrate that thresholds can be selected by defining a specificity or sensitivity value to obtain specified model performance. Appropriate cut-off can then be chosen according to purpose of use. The coefficients for the logistic regression models can be obtained from the odds ratios in Tables 1 and 2. In a clinical setting, the probability of an ADHD/ASD diagnosis can be calculated given a set of SDQ scores. The probability of a diagnosis can then be compared to the optimal threshold.

Goodman and Mullick [12] and Ullebo and colleagues [20] cautiously recommend use of the SDQ as a screening tool for childhood disorder and specifically ADHD/hyperkinetic disorder, Brøndbo and colleagues [25] caution against it. All these studies used well-validated scales measuring symptoms of ADHD. Our study used an outcome measure of parent-reported clinical diagnosis of disorder: as clinical assessments are highly variable and subject to local bias [49], our findings have no clinical application until replicated against standardised ADHD scales. It should be remembered that previous work on the algorithm [21] predicted against diagnoses made using a research instrument, while the current study uses parent-report of a clinical diagnosis; both studies report from a general population sample. For MCS, the PHD algorithm had low sensitivity at 30%, but a specificity of 98%.

The resulting LR model for autism shows that many types of difficulties may complicate the picture for a child with ASD. This is to be expected, as there is not a specific “autism spectrum” subscale that focuses on the core difficulties as there is with ADHD. Prosocial behaviour emerged as the strongest predictor of ASD, which again is not surprisingly as social impairments are core deficits. Furthermore, ASD diagnosis has been associated with the low scores on the prosocial subscale in other UK cohorts [50]. Our findings suggest that a range of other difficulties such as anxiety and conduct problems are likely to commonly co-occur with both ASD and ADHD, which, for those working with children who have these difficulties, echoes clinical experience. It is intriguing that ASD is not associated with conduct problems; indeed higher conduct problem ratings lower the odds of an ASD diagnosis. It may be that social difficulties inhibit the overt externalising behaviours covered by the SDQ, several of which require a social orientation towards others. Behaviour that challenges others among children with ASD often results from a failure to recognise or conform to social expectations and/or rigidity around routine or preferred activity, which may not be adequately tapped by the SDQ behaviour subscale. ASD was associated with enhanced emotional problems. These results concur with many studies that have found ASD to be associated with anxiety and depression [51]–[53]. Taking account of co-occurring symptoms is essential for any child with autism as it may have practical ramifications in terms of the type(s) of intervention required.

### Limitations

The current study used parent-report of clinical identification of ASD and ADHD by a doctor or another professional. This means that parents are likely to be well aware of symptoms of these conditions and may therefore be more likely to report them than a parent of a child with similar difficulties that have not been clinically highlighted. Furthermore, parents may have been over-inclusive in their interpretation of the question: inferring a positive answer in cases where ASD or ADHD was suggested by a health worker but not confirmed by further assessment. Clinician diagnoses themselves can be inaccurate if unguided by structured assessment [54].

In addition, the sample will contain other children with ASD and ADHD, and other disorders, as yet unrecognised [55], and research suggests that the unrecognised group may be in the majority [56]. It is beyond the scope of this article to comment on differentiation from other comorbid groups. As children with other disorders were mixed in with ‘general population’, the mean SDQ scores of the general population are likely to have been elevated and would serve to make the mean SDQ scores more similar to those of children with parent- reported ASD and ADHD. Thus, our detection of differences between children with a clinical diagnosis of ADHD and ASD is likely to be robust.

Other limitations relate to the ADHD group. First, we did not have access to pharmaceutical data, but evidence suggests treatment with methylphenidate may have improved symptoms of hyperactivity and may have led parents and teachers to under-report difficulties in any children with diagnoses who were taking medication [57]. Second, we did not have information on sub-type of ADHD. Ullebo and colleagues [20] found the SDQ was a good predictor for the combined ADHD subtype, but less informative for other subtypes. Third, seven years old is still early in life for clinical identification of ADHD [58], which may partially explain why the sensitivity of the PHD algorithm was so low in our study. It identified less than a third of the children with ADHD, a much poorer performance than witnessed in some other studies [20], [21]. At this age it is likely many cases are yet to be identified: assessment with a research-based diagnostic measure may have revealed different results.

Despite these limitations our results provide further evidence to suggest that the SDQ algorithm is a useful tool as an indicator of ADHD symptoms for research purposes. As the SDQ instrument is widely used in research studies already [6]–[13], bespoke cut-offs could be developed according to purpose of application to research. However, we do not currently recommend using the SDQ as a screening tool for either disorder in clinical practice due to the high number of false positives and limitations of case definition in our study. Should our findings be replicated against structured research assessments, they could be used by clinicians to identify children at risk of ADHD who warrant further assessment.

The study is part of a large and growing literature that demonstrates that ADHD symptoms are also relatively common in children and adults diagnosed with ASD and vice-versa [27]–[33]. It supports changes to DSM-5 dropping the exclusivity clause to allow dual diagnosis of ASD and ADHD, and suggests ICD criteria should follow suit: indeed dual diagnosis occurs in practice already. These results also suggest that for children with ASD, the presence of other co-occurring impairments in behaviour is likely to be the rule, not the exception. In this way the work contributes to the debate raised by Hattori et al. [33], [34] about whether the current diagnostic configuration for ADHD and autism are valid. The findings support the removal of exclusivity clauses in current revisions to DSM-5, and inform on-going debates about revisions to ICD-11.

## Supporting Information

Table S1 **Examples of scores over the threshold for ADHD model.** There are 1331 combinations of 3 SDQ scales (0–10) of which 928 combinations would produce a value over the threshold. The most frequent combinations in MCS are included for illustrative purposes.
(DOCX)

Table S2 **Examples of scores over the threshold for ASD model.** In MCS data there are 671 combinations which produce a value over the threshold. They are all unique. Table S2 gives 10 combinations which are over the threshold, for illustrative purposes.
(DOCX)
