# Supplementary material for: The Strengths and Difficulties Questionnaire as a Predictor of Parent-Reported Diagnosis of Autism Spectrum Disorder and Attention Deficit Hyperactivity Disorder
Source: PLoS One. 2013 Dec 3;8(12):e80247. doi: 10.1371/journal.pone.0080247 (PMC3848967; doi:10.1371/journal.pone.0080247)
Supplement: Table S1 — Examples of scores over the threshold for ADHD model. There are 1331 combinations of 3 SDQ scales (0–10) of which 928 combinations would produce a value over the threshold. The most frequent combinations in MCS are included for illustrative purposes. (DOCX) [file pone.0080247.s001.docx]

*Table S1:*

| SDQ Hyperactivity, teacher report | SDQ Hyperactivity, parent  report | SDQ Impact, parent  report | p | frequency in data |
| --- | --- | --- | --- | --- |
| 9 | 8 | 0 | 0.046 | 11 |
| 10 | 8 | 0 | 0.056 | 12 |
| 8 | 8 | 0 | 0.038 | 13 |
| 7 | 8 | 0 | 0.032 | 15 |
| 9 | 7 | 0 | 0.030 | 15 |
| 10 | 7 | 0 | 0.037 | 16 |
| 10 | 6 | 0 | 0.024 | 18 |
| 7 | 7 | 0 | 0.020 | 20 |
| 8 | 7 | 0 | 0.025 | 22 |
| 5 | 8 | 0 | 0.021 | 24 |
