# Supplementary material for: The Strengths and Difficulties Questionnaire as a Predictor of Parent-Reported Diagnosis of Autism Spectrum Disorder and Attention Deficit Hyperactivity Disorder
Source: PLoS One. 2013 Dec 3;8(12):e80247. doi: 10.1371/journal.pone.0080247 (PMC3848967; doi:10.1371/journal.pone.0080247)
Supplement: Table S2 — Examples of scores over the threshold for ASD model. In MCS data there are 671 combinations which produce a value over the threshold. They are all unique. Table S2 gives 10 combinations which are over the threshold, for illustrative purposes. (DOCX) [file pone.0080247.s002.docx]

*Table S2:*

| SDQ conduct problems, teacher report | SDQ Hyperactivity, teacher report | SDQ Impact, teacher  report | SDQ emotional problems, teacher report | SDQ Hyperactivity, parent  report | SDQ  Prosocial behaviour, parent  report | SDQ Impact, parent  report | p | frequency in data |
| --- | --- | --- | --- | --- | --- | --- | --- | --- |
| 0 | 2 | 0 | 5 | 7 | 8 | 2 | 0.030 | 1 |
| 4 | 9 | 2 | 0 | 9 | 6 | 0 | 0.030 | 1 |
| 0 | 9 | 1 | 0 | 9 | 9 | 1 | 0.030 | 1 |
| 0 | 1 | 0 | 4 | 9 | 10 | 3 | 0.030 | 1 |
| 3 | 6 | 6 | 1 | 2 | 8 | 0 | 0.030 | 1 |
| 0 | 6 | 2 | 6 | 4 | 10 | 1 | 0.030 | 1 |
| 2 | 6 | 3 | 3 | 5 | 7 | 0 | 0.030 | 1 |
| 0 | 8 | 2 | 1 | 6 | 7 | 0 | 0.030 | 1 |
| 5 | 5 | 3 | 3 | 9 | 7 | 0 | 0.030 | 1 |
| 0 | 4 | 1 | 4 | 10 | 8 | 0 | 0.030 | 1 |
